# Pelvic Floor Dysfunction in Postpartum Nepalese Women: An Observational Study

**DOI:** 10.31729/jnma.v64i293.9297

**Published:** 2026-01-31

**Authors:** Manisha Yadav, Sandesh Poudel, Shree Prasad Adhikari

**Affiliations:** 1 Paropakar Maternity and Women’s Hospital, Thapathali, Kathmandu, Nepal

**Keywords:** *pelvic floor dysfunctions*, *postpartum*, *primiparous*, *prevalence*, *urinary incontinence*, *vaginal birth*

## Abstract

**Introduction:**

Pelvic Floor Dysfunctions (PFD) have been strongly associated with vaginal birth and can significantly affect women’s quality of life. This study aimed to determine the prevalence of PFDs during the postpartum period and its impact on women’s daily activities.

**Methods:**

A hospital-based retrospective cross-sectional study was conducted among 747 primiparous women at 9-12 months postpartum, who had delivered term live baby from July to September 2021. The different components of PFD were evaluated using a Nepali translated version of PFDI-20 and the impact on sexual function by BFLUTS questionnaire. The primary outcome was women’s self-reported occurrence of urinary, prolapse, defecatory and sexual problems. The impact on women’s daily activities was secondary outcomes. Data were analyzed by Spearman’s Rho correlation coefficient.

**Results:**

Among 747 primiparous women, 194 (25.97%) had pelvic floor dysfunction. The mean age was 23.01±3.6 years, with 94 (48.45%) of PFD cases aged 20-24 years. Stress urinary incontinence was reported by 103 (53.09%), pelvic heaviness by 48 (24.74%), straining during defecation by 23 (11.86%), and dyspareunia by 54 (27.84%). Most women 137 (70.61%) reported no impact on daily life, and 18 (2.40%) were aware of pelvic floor muscle training.

**Conclusions:**

Pelvic floor dysfunction affected a notable proportion of primiparous women, with urinary, prolapse-related, colorectal, and sexual symptoms. Most affected women were young, and labour characteristics such as spontaneous onset, vaginal delivery, and shorter second stage were common.

## INTRODUCTION

Pelvic floor dysfunction (PFD) includes urinary incontinence (UI), pelvic organ prolapse, anal incontinence, perineal pain and sexual dysfunction and is strongly linked to childbirth-related pelvic floor weakness.^1,2^ Pregnancy induces anatomical and physiological changes that predispose women to PFD. With the advancement of pregnancy, high progesterone levels, acting on smooth muscle fibers and pelvic floor receptors, may reduce pelvic support; while the increasing mechanical pressure exerted by the gravid uterus places additional stain.^3^ During labour, the levator ani stretches to several times its resting length and even uncomplicated vaginal delivery can damage over 10% of muscle fibers.^4^ Repeated expulsive efforts may also injure the pudendal nerve, weakening urethral and pelvic support structures.^5,6^

The purpose of this study is to determine the prevalence of pelvic floor dysfunctions at 9-12 months postpartum period and to evaluate their impact on women’s daily activities and psychological well-being.

## METHODS

This was a retrospective cross sectional study conducted at Paropakar Maternity and Women’s Hospital, a tertiary care facility. The study population comprised 747 primiparous women who were 9-12 months postpartum and had delivered a live baby beyond 37 weeks of gestation between July and September 2021. Data for the study were collected from April to September 2022. The personal information of the women who met the inclusion criteria during the study period, were retrieved from the hospital data. Eligible participants were contacted via telephone and informed about the study. Verbal consent was obtained prior to enrolment. Women with diabetes mellitus (gestational or overt), intrauterine growth restriction, multiple pregnancy, pregnant during study period, history of Urinary incontinence in pre-pregnant state were excluded. Obstetric variables related to the index deliver - including nature of onset of labour, duration of second stage of labour, degree of perineal injury were retrieved from the hospital medical records. Onset of labour was noted as either spontaneous or induced. The second stage was defined and calculated as the interval between being fully dilated cervix the time of birth. The second stage was categorized as < 30 min, 30-60 min, > 60-90 min, 90>120 min. Perineal injury was graded from one to four degree.^7^ A telephone interview was conducted to assess current symptoms attributable to pelvic floor injury sustained during childbirth. During the interview, participants were asked about symptoms related to pelvic floor dysfunction and the extent to which these symptoms affected their day-to-day activities. Pelvic floor dysfunction was assessed using the Pelvic Floor Distress Inventory-20 (PFDI-20), which comprises three subscales: the Urinary Distress Inventory-6 (UDI-6) for urinary symptoms, the Pelvic Organ Prolapse Distress Inventory-6 (POPDI-6) for prolapse-related symptoms, and the Colorectal-Anal Distress Inventory-8 (CRADI-8) for colorectal symptoms.^8^ Participants were also questioned regarding sexual problems and the impact on sexual function was assessed using the sexual domain of the Bristol Female Lower Urinary Tract Symptoms (BFLUTS) questionnaire.^9^

This study is limited to assessing the presence of pelvic floor symptoms and the extent of patient-reported distress, without offering a qualitative or objective assessment of disease severity. As no validated Nepali versions of these questionnaires were available, the researcher developed a Nepali translation, which was reviewed by three urogynaecologists for linguistic accuracy and clinical relevance. A pilot face-to-face validation of the translated questionnaires was conducted among 10 participants prior to the study to ensure clarity, comprehensibility, and acceptability of the items. The primary outcome was women’s self-reported occurrence of UI, prolapse, defecatory and sexual problems. The impact of those PFD on women’s daily activities and psychological well-being are secondary outcomes. Frequency and percentage were used for the prevalence. The correlation between the variables was assessed using Spearman’s Rho correlation coefficient. Data analysis was conducted using IBM SPSS Statistics, version 26. Ethical approval for the study was obtained from the Institutional Review Committee (IRC) of Paropakar Maternity and Women’s Hospital, (Reference no. 63/319). Informed verbal consent was obtained from all participants, including permission to use their background information and medical records for research purposes. Participants were informed of their right to withdraw from the study at any time without any effect on their care. Privacy and confidentiality of all study participants were strictly maintained.

## RESULTS

Amongst 747 population included in the study 194 (25.97%) had PFD. The self-reported prevalence of specific disorders was as follows: urinary problems in 138 (18.47%), colorectal problems in 57 (7.63%), pelvic organ prolapse (POP)-related symptoms in 89 (11.91%), and sexual problems in 53 (7.09?%) participants. The mean age of the participants was 23.01±3.6 years. Participants were largely in the 20-24 years age group, accounting for 335 (44.80%) of the study population and 94 (48.45%) of cases with pelvic floor dysfunction (PFD). Labour onset was mainly spontaneous, recorded in 470 (62.91%) participants and 113 (58.24%) PFD cases. Spontaneous vaginal delivery (SVD) accounted for 626 (83.80%) of all deliveries and 168 (86.59%) among PFD cases and 113 (58.24%) cases of PFD noted among women with spontaneous onset of labor. Among instrumental deliveries, PFD was observed in 18 (9.27%) forceps-assisted deliveries and 8 (4.12%) vacuum-assisted deliveries . A second stage of labour lasting less than 30 minutes was observed in 384 (51.40%) participants and 132 (68.04%) PFD cases. Regarding perineal status, Grade 2 perineal tears accounted for 561 (75.10%) of participants and 152 (78.35%) of PFD cases. Knowledge of pelvic floor muscle training (PFMT) was reported by 18 (2.40%) participants, while PFMT practice was not reported (Table 1).

**Table 1:** Sociodemographic and obstetric characteristics

Demographic profile and obstetric variables	n (=747)	Cases with PFD (n=194)
Age (in years)	n(%)	n(%)
<20	106(14.19)	23(11.85)
20-24	335(44.84)	94(48.45)
25-29	302(40.42)	77(39.69)
30-35	4(0.53)	-
>35	-	-
Nature of Onset of Labour		
Spontaneous	470(62.91)	113(58.24)
Induced	277(37.09)	81(41.75)
Mode of Delivery		
SVD	626(83.80)	168(86.59)
Instrumental: Vaccuum	65(8.70)	8(4.12)
Forcep	56(7.49)	18(9.27)
II stage of labour		
<30	384(51.40)	132(68.04)
30-60	145(19.41)	8(4.12)
60-90	32(4.28)	17(8.76)
90≥120	186(24.89)	37(19.07)
Perineal Tear		
Grade 1	153	34(17.52)
Grade 2	561	152(78.35)
Grade 3	22	8(4.12)
Grade 4	11	-
PFMT knowing status	18	-
PFMT Doing status	-	-

PFMT = Pelvic floor muscle training

**Table 2:** Spearman’s Rho Correlation Coefficient Analysis Matrix (n=194).

Nature of onset of labor	Mode of delivery	Duration of second stage of labour	Perineal tear	Urinary symptoms	Colorectal symptoms	POP symptoms
Nature of onset of labor						
Mode of delivery	-.082						
Duration of second stage of labor	.096	.181					
Perineal tear	-.081	.109	-.023				
Urinary symptoms	.024	.027	-.051	-.081			
Colorectal symptoms	-.003	-.037	-.124	-.048	.292		
POP symptoms	-.016	-.050	-.164	-.097	.453	.283	

**Figure 1:**
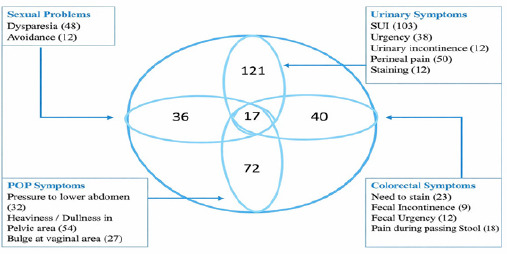
Distribution of urinary, colorectal, POP and sexual symptoms (n=194).

Among the 194 women with PFD, 17 (8.76%) experienced symptoms across all four domains (Figure 1). Stress urinary incontinence was reported by 103 (53.09%) participants. Straining during defecation was reported by 23 (11.86%) participants. In the pelvic organ prolapse domain, heaviness or a dull pelvic sensation was reported by 48 (24.74%) participants. Dyspareunia was reported by 54 (27.84%) participants reporting sexual problems. Spearman’s correlation analysis showed an linear relationship between duration of the second stage of labour and mode of delivery (r = 0.181), and between duration of the second stage of labour and nature of onset of labour (r = 0.096), (Table 2). Overall, 137 (70.61%) of women reported that their symptoms had no impact on daily activities or interpersonal relationships.(Table 1).

## DISCUSSION

Pelvic floor dysfunction represents a significant and multifaceted health issue, encompassing conditions like urinary incontinence, pelvic organ prolapse, fecal incontinence and pelvic-perineal pain syndrome. It is a global health problem affecting millions of women worldwide. Many women often perceive those conditions as social stigma and regard it as the normal consequences of childbirth, leading to under-reporting of symptoms. PFD can have profound psychosocial consequences, including feelings of shame, humiliation, anxiety, depression and social isolation. Among these, sexual dysfunction is often the most distressing outcome.

Urinary incontinence is among the most prevalent pelvic floor dysfunctions encountered during pregnancy and postpartum period. It is defined as the complaint of involuntary loss of urine, affecting 3% to 40% of women after childbirth.^10,11^ The condition is attributed to anatomical and physiological changes of pregnancy: enlarged uterus, hormone fluctuation, elevated glomerular filtration rates, temporary alteration in the urethrovesical angle. While often transient and resolving withing 3 months postpartum; UI can persist or emerge as a de novo condition.^12,13^ Unlike antenatal incontinence, postpartum incontinence is mainly attributed to delivery related injuries that involves bladder, pelvic nerve, muscles or urethral support structures. Symptoms within three months postpartum may be reflective of the acute injury sustained at delivery and often reversible. However, those symptoms persisting beyond three months postpartum have an increased risk of long-term symptoms.^12,13^ Other forms of PFD are attributed to similar underlying mechanism. This study found over a quarter of primiparous women experienced over one form of PFD in postpartum. In the present study, the overall prevalence of PFD among primiparous women was 25.97%, with urinary symptoms being the most frequent, followed by pelvic organ prolapse-related, colorectal and sexual symptoms (18.5%, 11.9%, 7.6%, and 7.1%, respectively). In contrast, Suemitsu et al. reported a prevalence of 73.6% (156 of 212 participants) when including both primiparous and multiparous women, with urinary symptoms also predominating. The discrepancy that may be due to differences in study population, as their research included multiparous women who have a cumulative risk from repeated obstetric trauma. Their study also employed the PFDI-20 questionnaire as the assessment tool.^14^ Contrary to the common understanding that PFD risk increases with age, our study showed a higher prevalence in the 20-24-year age group. This highlights that obstetric trauma might be a primary etiological factor in young primiparous women, independent of age and parity related connective tissue changes. The nature of onset of labour and mode of delivery did not correlate with urinary, colorectal and prolapse symptoms in our study. Pregnancy itself, through its mechanical pressure, the anatomical and physiological changes, stretching and compression of the pelvic nerves, can weaken both the fascial structure and the connective tissue supporting the pelvic floor. Those factors contribute to pelvic floor weakening regardless of whether labor is spontaneous or induced, or whether delivery is spontaneous or instrumental. Nevertheless, cesarean section has been shown to be associated with a lower incidence of postpartum PFD than vaginal delivery.^13^ Women were more likely to report urinary incontinence after vaginal delivery than after cesarean sections at six years postpartum.^15^ Similar to our findings, other studies have reported persistent SUI to be more common following vaginal than caesarean delivery.^16^ In the current study, the duration of the second stage of labour showed no correlation with urinary symptoms but had a weak, negative correlation with colorectal and prolapse symptoms. This may imply that the maximum degree of stretching during fetal expulsion is more critical than the total duration. A study by Ahlund et al.^17^ also found no association between the duration of the second stage and urinary incontinence. However, an observational study concluded duration of second stage of labour ≥ 90mins being the risk factors for postpartum SUI.^18^ A recent systematic review and meta-analysis identified several risk factors for postpartum SUI, including vaginal delivery, advanced maternal age, high body mass index (BMI), excessive gestational weight gain, diabetes, episiotomy, forceps delivery and prenatal urinary incontinence.^19^. Contrary to these findings, our study found PFD to be more common among younger women and those with spontaneous vaginal delivery. Consistent with Wang et al.,^19^ episiotomy was also associated with PFD symptoms in our cohort. A majority of participants (70.61%) reported that their symptoms had no significant impact on daily activities or interpersonal relationships. However, nearly one-third of women with PFD reported adverse psychological effects such as depression, exhaustion and anxiety, along with concerns about sexual relationships. Whoolhouse et al. ^20^ similarly observed that postpartum urinary incontinence can lead to fatigue, back pain, sexual dysfunction and mood disturbances. Another study found that postpartum depression was significantly higher among women with urinary incontinence, particularly those aged 15-19 or >40 years, from low-income groups or delivered by caesarean section .^21^ Therefore, early and accurate diagnosis of PFD in postpartum women, coupled with ongoing follow-up and supportive counselling, is essential to improve quality of life. Awareness of pelvic floor muscle exercises was extremely low in our study, only 2.3% of women were aware, and none practiced them. Many believed these exercises were indicated only after vaginal hysterectomy. Evidence suggests that regular pelvic floor muscle training in postpartum women significantly improves pelvic floor function. ^22^ Hence, healthcare providers should prioritize early identification of symptoms, education on pelvic floor exercises and motivation to practice them correctly to reduce the risk of PFD development.

This study comprehensively assessed all domains of pelvic floor function using a Nepali translation of validated questionnaires, rather than focusing on a single aspect. Importantly, sexual function, a vital but often neglected component of postpartum health, was also evaluated.

The present study has several limitations. It was a retrospective single-centered hospital based study and potential unmeasured confounders may have influenced the results. The assessment tool used was not validated one. Key variables such as body mass index (BMI), a known strong determinant of PFD—were unavailable. Information on ethnicity and educational status was also lacking, as there is association between neonatal birth weight and PFD. Moreover, participants were followed up at one year postpartum only; longer-term follow-up is necessary for more conclusive results.

## CONCLUSION

Pelvic floor dysfunction was identified among a considerable proportion of primiparous women, with symptoms spanning urinary, pelvic organ prolapse-related, colorectal, and sexual domains. The condition was observed largely in young women, particularly those in early adulthood. Spontaneous onset of labour, spontaneous vaginal delivery, shorter duration of the second stage of labour, and Grade 2 perineal tears characterized many affected participants. Stress urinary incontinence, pelvic heaviness, straining during defecation, and dyspareunia emerged as prominent symptom patterns, and some women experienced symptoms across multiple pelvic floor domains. Despite the presence of these symptoms, many women reported no perceived impact on daily activities or interpersonal relationships. Knowledge and practice of pelvic floor muscle training were limited.

## Data Availability

The data are available from the corresponding author upon reasonable request.
